# Supplementary material for: GSDM gene polymorphisms regulate the IgE level in asthmatic patients
Source: PLoS One. 2022 Oct 6;17(10):e0274951. doi: 10.1371/journal.pone.0274951 (PMC9536611; doi:10.1371/journal.pone.0274951)
Supplement: Attachment — Submitted filename: Response to Reviewers.docx [file pone.0274951.s002.docx]

GSDM gene polymorphisms regulate the IgE level in asthmatic patients

reference: PONE-D-21-33691

Dear Editor,

Accompanying this letter are the revised version of the manuscript with all changes needed, and response to reviewers’ comments. We would like to thank the respected reviewers again for taking the time and effort to read the revised manuscript, make some corrections, and for their comments and suggestions which will definitely improve the quality of the paper. We have addressed most of the points, comments, and suggestions made by the reviewers and hope the manuscript should come to a happy ending.

We thank you for your consideration and look forward to hearing from you soon.

reference: PONE-D-21-33691

Title: GSDM gene polymorphisms regulate the IgE level in asthmatic patients

PLOS One Reports: Amer Imraish, Tuqa Abu-Thiab, Tareq Alhindi, Malek Zihlif

Reviewers' comments:

Reviewer 1

This study investigated the association of SNP of GSDMA (rs7212938, T/G) and GSDMB (rs7216389, T/C) in Jordanian population with total IgE levels in serum of asthmatic children and adult subjects. It was found that GSDMB SNP increased susceptibility to asthma development in childhood in a way independent of IgE release, and no significant association was found between GSDMA and IgE levels in both child and adult asthmatic patients. The topic is useful and the conclusion is reliable. However, a minor modify could be completed.

1. In the method section

- Please state the age range of the patients and controls.

The age range was added to the Table 1.

-Please state the cat no. of the used ELISA kit

The following was added to the revised manuscript in the method part

cat. no. RE59061; IBL International, Corp.

2. in the results section

-Gasdermin A (GSDMA) and gasdermin B (GSDMB) genes are located at chromosome 17, and variants of these genes are confirmed to increase susceptibility to asthma phenotypes in children (please add reference)

The reference has been added.

- Please state which clinical respiratory parameters were measured.
the following was added to the results section:

[forced expiratory volume (FEV1) and forced vital capacity (FVC)].

Reviewer #2:

Based on an association between the Gasdermin A and Gasdermin B polymorphisms and susceptibility to adult and childhood asthma in the Jordanian population, the association between GSDMA (rs7212938, T/G) and GSDMB (rs7216389, T/C) at locus 17q12-21 and level of IgE immunoglobulin antibody in child and adult Jordanian asthma patients has been tested.

This study confirmed that there is a significant association between GSDMB genetic SNP (rs721638) and IgE levels in asthma patients in Jordanian population. Overall, the results are meaningful. Minor concern is below.

1. The author need to add the forward and the reverse primers sequences

The primers sequences were added to the revised manuscript in the method part

2. The author need to state the name of normality test that have been used

The following was added to the method part in the revised manuscript

The normality of data was determined using a Pearson normal distribution curve, and the results showed that all data were normally distributed.

3. When did the Peripheral blood specimens were collected from patients? at the acute attack or stable stage of asthma attack?

Thanks much for your interesting comments. For the first point, the blood samples were collected in stable stage. At least not under exacerbation that needed oral cortisone.
