# Supplementary material for: GSDM gene polymorphisms regulate the IgE level in asthmatic patients
Source: PLoS One. 2022 Oct 6;17(10):e0274951. doi: 10.1371/journal.pone.0274951 (PMC9536611; doi:10.1371/journal.pone.0274951)
Supplement: Attachment — Submitted filename: Response to Reviewers.docx [file pone.0274951.s003.docx]

GSDM gene polymorphisms regulate the IgE level in asthmatic patients

reference: PONE-D-21-33691

Dear Editor,

Accompanying this letter are the revised version of the manuscript with all changes needed, and response to reviewers’ comments. We would like to thank the respected reviewers again for taking the time and effort to read the revised manuscript, make some corrections, and for their comments and suggestions which will definitely improve the quality of the paper. We have addressed most of the points, comments, and suggestions made by the reviewers and hope the manuscript should come to a happy ending.

We thank you for your consideration and look forward to hearing from you soon.

reference: PONE-D-21-33691

Title: GSDM gene polymorphisms regulate the IgE level in asthmatic patients

PLOS One Reports: Amer Imraish, Tuqa Abu-Thiab, Tareq Alhindi, Malek Zihlif

Reviewers' comments:

Reviewer 1

This study investigated the association of SNP of GSDMA (rs7212938, T/G) and GSDMB (rs7216389, T/C) in Jordanian population with total IgE levels in serum of asthmatic children and adult subjects. It was found that GSDMB SNP increased susceptibility to asthma development in childhood in a way independent of IgE release, and no significant association was found between GSDMA and IgE levels in both child and adult asthmatic patients. The topic is useful and the conclusion is reliable. However, a minor modify could be completed.

1. In the method section

- Please state the age range of the patients and controls.

The age range was added to the Table 1.

-Please state the cat no. of the used ELISA kit

The following was added to the revised manuscript in the method part

cat. no. RE59061; IBL International, Corp.

2. in the results section

-Gasdermin A (GSDMA) and gasdermin B (GSDMB) genes are located at chromosome 17, and variants of these genes are confirmed to increase susceptibility to asthma phenotypes in children (please add reference)

The reference has been added.

- Please state which clinical respiratory parameters were measured.
the following was added to the results section:

[forced expiratory volume (FEV1) and forced vital capacity (FVC)].

Reviewer #2:

Based on an association between the Gasdermin A and Gasdermin B polymorphisms and susceptibility to adult and childhood asthma in the Jordanian population, the association between GSDMA (rs7212938, T/G) and GSDMB (rs7216389, T/C) at locus 17q12-21 and level of IgE immunoglobulin antibody in child and adult Jordanian asthma patients has been tested.

This study confirmed that there is a significant association between GSDMB genetic SNP (rs721638) and IgE levels in asthma patients in Jordanian population. Overall, the results are meaningful. Minor concern is below.

1. The author need to add the forward and the reverse primers sequences

The primers sequences were added to the revised manuscript in the method part

2. The author need to state the name of normality test that have been used

The following was added to the method part in the revised manuscript

The normality of data was determined using a Pearson normal distribution curve, and the results showed that all data were normally distributed.

3. When did the Peripheral blood specimens were collected from patients? at the acute attack or stable stage of asthma attack?

Thanks much for your interesting comments. For the first point, the blood samples were collected in stable stage. At least not under exacerbation that needed oral cortisone.

Response to Editor

#1. Please describe strengths and limitations section.

Experimental limitations

Our results reflected the patients and controls who are included in the study; although our sample size is considered convenient, increasing the sample size could alter the patient/control ratio of each SNP and provide more accurate results.

#2. Funding: With what research funds did the author conduct this analysis? Was it carried out at your own expense?

This research was funded from the deanship of academic research at the University of Jordan.

#3. Identify the exact inclusion criteria for 392 participants.

The chief inclusion criterion for all groups was ethnicity. Participants were exclusively drawn from the same ethnic group (namely Arabs) in order to minimize population stratification. Ethnicity was determined by self-report.

Exclusion criteria were a present or past history of asthma and asthma-like symptoms. None of the patients had received specific immunotherapy for allergic conditions.

None of the patients had any autoimmune disease. The control subjects were all symptoms free, had no history of atopic disorders, and had normal IgE level. All patients and control subjects have signed an informed consent.

#4. What is the definition of asthma?

The following has been added to the manuscript:

Asthma is an immunological abnormality characterized by a chronic airway constriction that develop both in adults and children. The hallmarks of the disease are the airway obstruction, wheezing, accumulation of IgE antibodies in response to inhaled allergens and broncho-hyperresponse.

#5. Describe the IgE sampling in the method.

The following has been added to the manuscript:

Samples of peripheral blood of all participants were collected in EDTA tubes, and centrifuged at 1,500 xg for 10 minutes at 4 ^ͦ^C, then the serum supernatant was aspirated in a clean collection tube and stored at -80 ^ͦ^C until being used.

#6. Describe the IgE mean level in the Table 1.

The mean (range) of total IgE levels in patients and control adult subjects was 134.14 IU/mL and 17.22 IU/mL, respectively, and in patients and control children was 172.4 IU/mL and 21.25 IU/mL, respectively. According to the kit ranges, subjects with IgE level below 100 IU/mL were considered normal IgE level.

#7. Did the author only conduct baseline surveys on age, IgE level, and asthma? This level of investigation cannot provide a faithful correction to the selection bias.
we have recorded other parameter such as forced expiratory volume (FEV1) and forced vital capacity (FVC)]

#8. In statistical section, the authors have to mention the statistical guideline such as <https://doi.org/10.54724/lc.2022.e3>

The following has been added to the manuscript:

GraphPad Prism 7.0 software (GraphPad Software, La Jolla, CA) was used for statistical analysis of the collected data. The normality of data was determined using a Pearson normal distribution curve, and the results showed that all data were normally distributed. After passing normality test, and According to GraphPad prism software guidelines, student t-test was used to perform comparisons, where statistical significance has been considered with P <0.05 (Delacre, et al. 2017). Results are presented as mean± S.E.M.

#9. I cannot believe that 300 people were not excluded from the study. Usually, 10% exclusion is made in large-scale international studies.

The study prospective study, in which we included the those patients who is fitted in the inclusion criteria. For example the patient has to be confirmed to have asthma from the clinician to be included and further more the asthma has to be followed by the respiratory test for confirmation . There are many patients that visit the respiratory clinic at the University of Jordan hospital and not all of them were included. In short it is a case control study.
